# Formulative Study and Intracellular Fate Evaluation of Ethosomes and Transethosomes for Vitamin D3 Delivery

**DOI:** 10.3390/ijms22105341

**Published:** 2021-05-19

**Authors:** Manuela Costanzo, Elisabetta Esposito, Maddalena Sguizzato, Maria Assunta Lacavalla, Markus Drechsler, Giuseppe Valacchi, Carlo Zancanaro, Manuela Malatesta

**Affiliations:** 1 Department of Neurosciences, Biomedicine and Movement Sciences, University of Verona, I-37134 Verona, Italy; manuela.costanzo@univr.it (M.C.); mariaassunta.lacavalla@univr.it (M.A.L.); carlo.zancanaro@univr.it (C.Z.); 2 Department of Chemical, Pharmaceutical and Agricultural Sciences, University of Ferrara, I-44121 Ferrara, Italy; elisabetta.esposito@unife.it (E.E.); sgzmdl@unife.it (M.S.); 3 Bavarian Polymer Institute (BPI) Keylab Electron and Optical Microscopy, University of Bayreuth, D-95440 Bayreuth, Germany; markus.drechsler@uni-bayreuth.de; 4 Department of Neurosciences and Rehabilitation, University of Ferrara, I-44121 Ferrara, Italy; vlcgpp@unife.it; 5 Animal Science Department, Plants for Human Health Institute, NC Research Campus, NC State University, Kannapolis, NC 28081, USA

**Keywords:** cell culture, cholecalciferol, cryogenic transmission electron microscopy, in vitro test, light microscopy, lipid nanocarriers, transmission electron microscopy

## Abstract

In this pilot study, ethosomes and transethosomes were investigated as potential delivery systems for cholecalciferol (vitamin D3), whose deficiency has been correlated to many disorders such as dermatological diseases, systemic infections, cancer and sarcopenia. A formulative study on the influence of pharmaceutically acceptable ionic and non-ionic surfactants allowed the preparation of different transethosomes. In vitro cytotoxicity was evaluated in different cell types representative of epithelial, connective and muscle tissue. Then, the selected nanocarriers were further investigated at light and transmission electron microscopy to evaluate their uptake and intracellular fate. Both ethosomes and transethosomes proven to have physicochemical properties optimal for transdermal penetration and efficient vitamin D3 loading; moreover, nanocarriers were easily internalized by all cell types, although they followed distinct intracellular fates: ethosomes persisted for long times inside the cytoplasm, without inducing subcellular alteration, while transethosomes underwent rapid degradation giving rise to an intracellular accumulation of lipids. These basic results provide a solid scientific background to in vivo investigations aimed at exploring the efficacy of vitamin D3 transdermal administration in different experimental and pathological conditions.

## 1. Introduction

The role of vitamin D (VD) on health is well established; indeed, it directly affects lymphocytes functions and cytokines secretion, exerting in this way anti-inflammatory properties [1]. The exposure of skin to ultraviolet radiation induces the synthesis of the steroid hormone cholecalciferol from 7-dehydrocholesterol. Many dermatological disorders, systemic infections, and cancers can be related to low VD levels [2]. Since cholecalciferol (or vitamin D3, VD3) and other VD analogues (e.g., ergocalciferol, or vitamin D2) are characterized by anti-proliferative and pro-differentiating effects, they have been demonstrated to be highly efficient in the treatment of several skin conditions, including psoriasis vulgaris [3]. In addition, the synthesis of VD in the skin plays an important role for the prevention of many diseases, such as ultraviolet B-induced melanoma [3]. Furthermore, recent findings demonstrated that VD deficiency influences muscle mass and responses [4]. Indeed, VD supplementation can limit sarcopenia and improve muscle performance in elder people [5,6]. Notwithstanding some dietary supplements of VD can be effective in reducing its deficiency, further VD topical application could represent a strategy to counteract skin disease and/or improve muscular function, especially in the case of people suffering from VD deficiency associated with nutrition problems, aging, and hepatic or renal disorders.

In this context, the possibility of delivering VD through the skin either to improve cutaneous conditions or to reach internal organs represents an important target. The difficulty in skin administration of highly lipophilic active ingredients such as VD is represented by the impossibility of crossing the barrier of the *stratum corneum*. Indeed, when administered in cream formulations, VD tends to deposit on the skin surface due to its affinity with the vehicle [7,8]. In order to overcome this drawback and to promote VD permeation through the skin, despite its physicochemical characteristics, specialized transdermal delivery systems with penetration enhancement properties are required. In this regard, recently, a liposomal formulation of VD was proposed for cutaneous application in the treatment of photoaging [9], while many commercial oral nutritional supplements declare the presence of liposome containing VD in their composition. Liposomes are vesicular systems mainly constituted of phospholipids, such as phosphatidylcholine (PC), and water, representing the first generation of nanosystems for drug encapsulation and transdermal delivery. Their peculiar composition results in the formation of multilamellar vesicular systems, characterized by a supramolecular structure resembling the three-dimensional organization of the epidermis *stratum corneum*. Notwithstanding their well-known potential as a drug delivery system, liposomes present instability problems that can lead to sedimentation, rupture of vesicles and leakage of the encapsulated drug [10].

To solve these drawbacks, new generations of lipid-based nanosystems have been proposed, such as transferosomes and ethosomes (ET) [11,12,13]. ET are vesicular systems made of PC, ethanol (20–45%) and water, characterized by a higher thermodynamic stability and loading capacity with respect to liposomes. Indeed, the presence of ethanol stabilizes vesicles, improves the solubility of lipophilic drugs and confers to ET a particular softness [9,10,11]. At the same time, ethanol associated to PC helps to open ways through the *stratum corneum* barrier, thus promoting ET passage through the skin [14,15]. Indeed, some studies have demonstrated the capability of ET to cross different biological membranes [16,17,18]. Moreover, recent studies have been performed to improve the transdermal potential of lipid nanosystems by modifying the vesicle compositions [19,20]. Particularly in the case of transferosomes and transethosomes, the addition as edge activators of surfactants to the phospholipid matrix has been proposed, thus modifying the vesicle deformability and improving transdermal penetration once applied on the skin [20].

The present work is a pilot study aimed at investigating the suitability of ET and transethosomes as nanocarriers to deliver VD3. Particularly, a formulative study has been conducted investigating the influence of pharmaceutically acceptable ionic and non-ionic surfactants in the preparation of different transethosomes. Vesicle size distribution, morphology, VD3 entrapment efficiency and relative vesicle deformability were determined. The cytotoxicity of ET and transethosomes in vitro was assessed on cells of different histological lineages that may be found in the skin; namely, keratinocytes (as the epithelial cells composing the epidermis), fibroblasts (as the typical cells in the connective tissues) and myoblasts (as cells of the skeletal muscle tissue). The selected nanocarriers were further investigated for their biological suitability by monitoring their uptake and intracellular fate in the three cell types at light and transmission electron microscopy.

## 2. Results

### 2.1. Preparation of Ethosomes and Transethosomes

In order to find vehicles suitable for non-invasive administration of VD3 to the skin and muscle tissue, biocompatible transdermal nanosystems were investigated. Particularly, ET were considered, being phospholipid-based vesicular systems containing high amounts of ethanol, characterized by penetration enhancer properties. In addition, the influence of non-ionic and ionic surfactants as edge activators added to the ethosomal composition was considered. Particularly, ET made of PC ethanol solution and water (30:70 *v*/*v*) were produced, while the addition of polysorbate 80 (T80), sodium cholate (SC) or dimethyldidodecylammonium bromide (DD) to the PC ethanol solution resulted in transethosomes, respectively, named TET, SCET or DET, as reported in Table 1. A cold method enabled one to spontaneously and rapidly obtain milky dispersions in the case of ET and translucent dispersions in the case of TET, SCET or DET.

### 2.2. Size Distribution

In order to shed light on the size distribution of ET, TET, SCET and DET, and to select the formulations suitable to transdermally deliver VD3, a dynamic light scattering analysis was performed by photon correlation spectroscopy (PCS). Vesicle mean diameters expressed as Z Average and dispersity indexes are reported in Table 2. Z Average values ranged between 111 and 277 nm, while dispersity index values were below 0.2, indicating a homogeneous size population [21]. The mean diameter of ET vesicle was around 200 nm. With regard to transethosomes, the smallest mean diameter was achieved in the case of DET, while the largest mean diameter was found in SCET. The use of the non-ionic surfactant T80 resulted in TET vesicles whose mean diameter was 186 nm. In order to choose vesicles with sizes compatible with transcutaneous administration, ET, TET and DET were selected.

### 2.3. Cytotoxicity of Ethosomes and Transethosomes

Treatment with DET induced a massive cell death and consequent detachment from the substrate at all the concentrations tested already after 2 h incubation; therefore, no MTT was performed. Due to this high cytotoxicity, DET were excluded from the further experiments.

**Figure 1:**
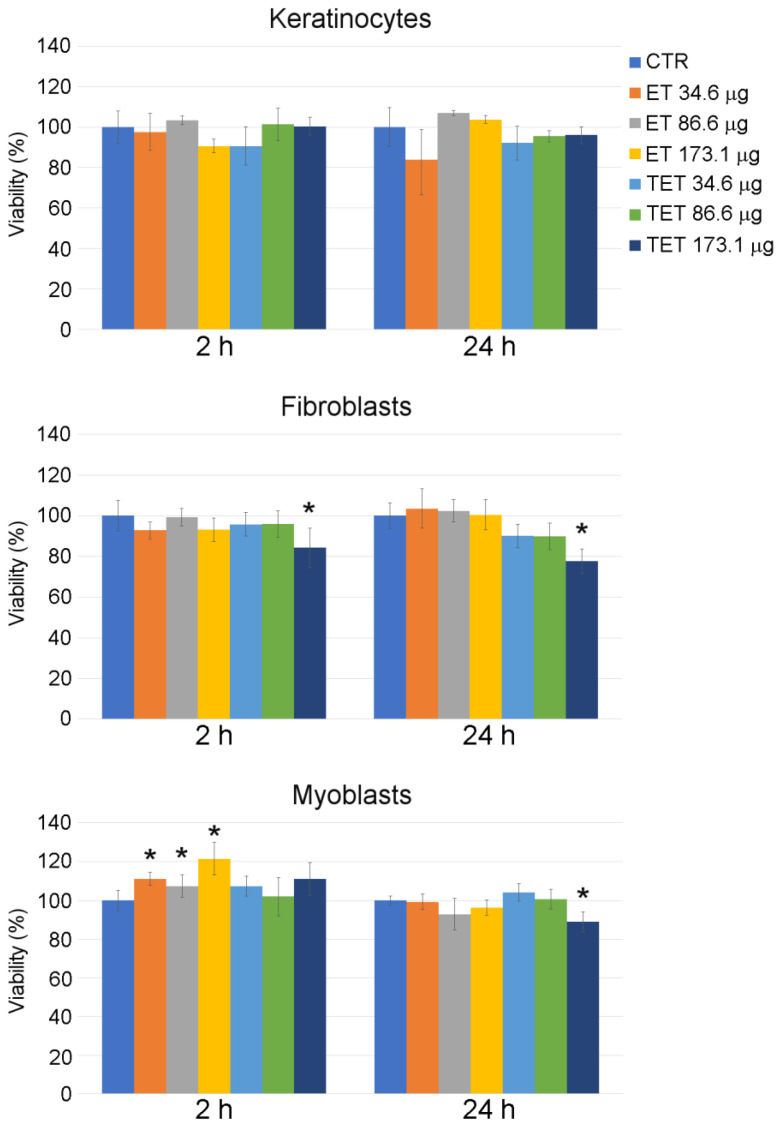
Effect of ET and TET on cell viability of cultured keratinocytes, fibroblasts and myoblasts, as measured by MTT assay. Histograms show the mean percentage value ± s.d. of cell viability after 2 h and 24 h of incubation with ET and TET at different concentrations. CTR: control (untreated) cells. * *p* < 0.05.

On the contrary, ET and TET proved to be safe for all the cell types at all time points considered up to the PC concentration of 86.6 µg (Figure 1). The concentration of 173.1 µg increased cell death in myoblasts and fibroblasts and was thus excluded from the subsequent experimentation.

ET incubation for 2 h resulted in an increase in MTT signal in myoblasts, according to previous findings that PC-based nanoparticles may increase cell viability by the activation of the MEK-ERK1/2 pathway or cell metabolism [22].

### 2.4. Preparation and Characterization of Vitamin D3 Containing Ethosomes and Transethosomes

The viability test enabled one to select ET and TET as non-toxic formulations to be loaded with VD3. To this aim, the drug was added to the PC ethanol solution, before water addition, resulting in ET-VD3 and TET-VD3 formulations, whose compositions are reported in Table 1.

**Figure 2:**
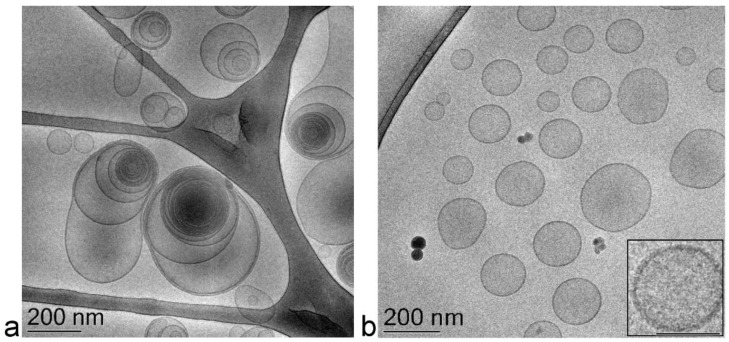
Cryo-TEM images of ET-VD3 (**a**) and TET-VD3 (**b**). The bar corresponds to 200 nm in panels (**a**) and (**b**), 100 nm in the inset of panel (**b**).

The PCS analysis of size distribution revealed that adding the drug slightly increased the vesicles’ mean diameter; namely, 40 nm in the case of ET-VD3 and 20 nm in the case of TET-VD3 (Table 2), without affecting the dispersity index. Vesicle morphology was visualized by cryogenic transmission electron microscopy (cryo-TEM): ET-VD3 appeared as spherical or ovoid vesicles with a multilamellar structure, typical of the PC double-layer organization (Figure 2a), while TET-VD3 appeared as unilamellar vesicles (Figure 2b). These different morphological features suggest that, in the presence of VD3, T80 disorganizes the multilamellar structure of PC, possibly because of an interaction between T80 and PC, arranging their polar heads towards the aqueous phase and the hydrophobic tails inside the double layer of the vesicles. T80 is supposed to sterically hamper the multilayer organization of PC in the presence of VD3, while retaining the vesicle double layer (inset in Figure 2b).

Both ET-VD3 and TET-VD3 were able to completely entrap the drug within the vesicles, as determined by ultracentrifugation, disaggregation and HPLC analyses. Indeed, VD3 was entirely associated to the PC phase, resulting in 100% EC values (Table 2) due to VD3 high solubility in ethanol. Notably, the cold method of preparation avoids thermal stresses that might possibly result in drug degradation.

### 2.5. Cytotoxicity of Vitamin D3 Containing Ethosomes and Transethosomes

**Figure 3:**
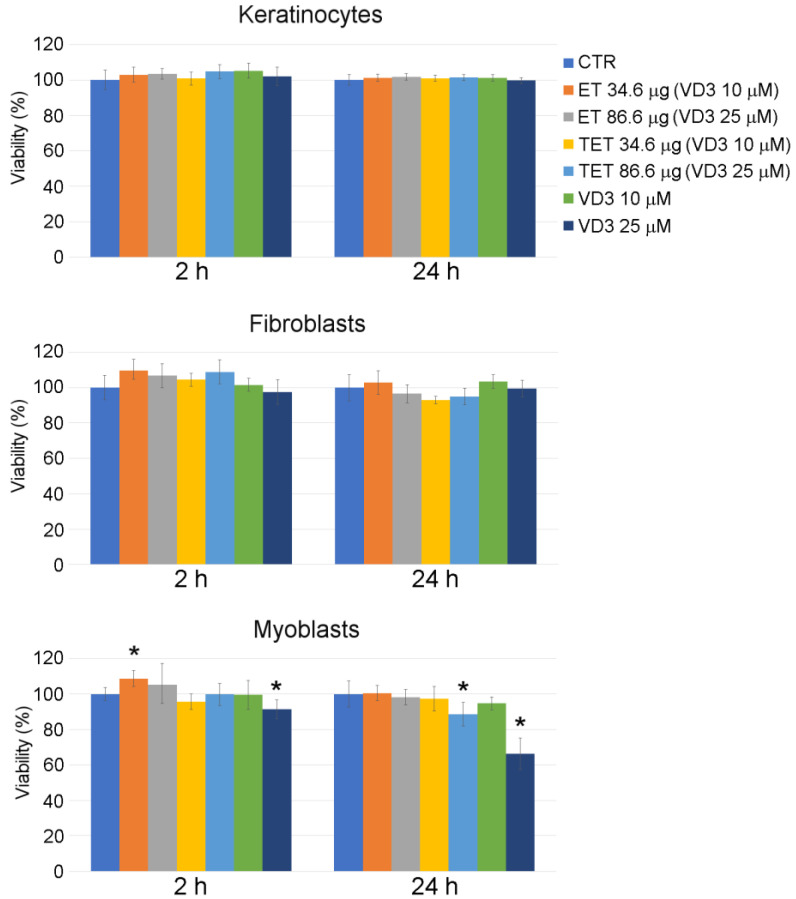
Effect of ET-VD3, TET-VD3 and VD3 on cell viability of cultured keratinocytes, fibroblasts and myoblasts, as measured by MTT assay. Histograms show the mean percentage value ± s.d. of cell viability after 2 h and 24 h of incubation with nanocarriers or VD3 as in at different concentrations. * *p* < 0.05.

After entrapping VD3 into ET and TET formulations previously found to be safe (i.e., PC concentrations of 34.6 μg and 86.6 μg), both nanocarriers confirmed their safety at all time points, except TET-VD3 25 μM, which increased cell death in myoblasts after 24 h incubation (Figure 3). A 25 μM VD3 solution increased cell death after both 2 h and 24 h incubation.

Again, incubation for 2 h with ET resulted in an increase in MTT signal in myoblasts, although only at the PC concentration of 34.6 μg/mL. In this case, the highly metabolizing and proliferative action of PC [22] was probably counterbalanced by the anti-proliferative effect of VD3 [23].

### 2.6. Deformability Study

The process of ET and TET penetration through the skin is related to the ethanol capability to fluidize the lipid domain of the *stratum corneum*, and to the vesicle peculiar capability to deform [19]. To compare vesicle deformability, ET, TET, ET-VD3 and TET-VD3 were subjected to extrusion tests, measuring the variation of mean diameter before and after the extrusion process. The relative deformability values are reported in Table 2. The deformability values of TET and TET-VD3 were almost double with respect to ET and ET-VD3, suggesting that the presence of T80 increases vesicle softness, both in the presence and in the absence of VD3.

### 2.7. Stability Evaluation

**Figure 4:**
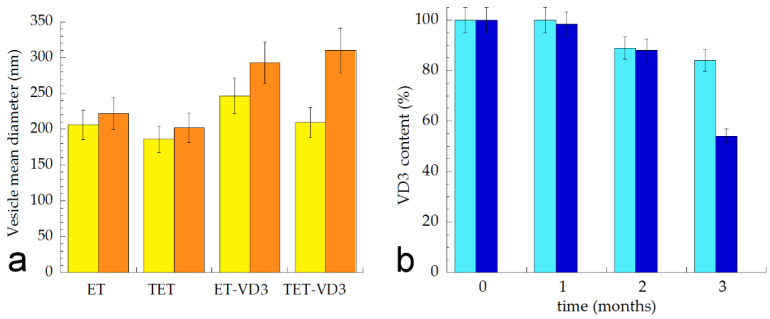
(**a**) Mean diameter variation for the indicated ET and TET. Mean diameters were measured by PCS after 0 (yellow) and 3 months (orange) from production and expressed as Z average. Data are the mean of three determinations on different samples; (**b**) Variation of VD3 content in ET-VD3 (light blue) and TET-VD3 (blue) determined up to 3 months from production. Bars indicate s.d.

Vesicle mean diameters and dispersity indexes were evaluated by PCS after 3 months, storing samples in the light at 22 °C in order to check their size stability. As reported in Figure 4a, vesicle size underwent a slight increase in the case of ET and TET (≈20 nm) after 90 days from the production, while a higher increase was detected in the case of VD3-containing vesicles, especially for TET-VD3, displaying a 100 nm increase. Dispersity indexes did not increase, being always below 0.2, suggesting a homogeneous size distribution. In order to compare the effectiveness of ET-VD3 and TET-VD3 in controlling drug degradation, VD3 entrapment was evaluated within 3 months from ET-VD3 and TET-VD3 preparation. Notably, ET and TET firmly entrapped VD3 up to 1 month; afterwards VD3 content decreased, especially in the case of TET-VD3, being 52% after 90 days of storage, suggesting a firmer retention of VD3 within the multilamellar structure of ET with respect to the TET unilamellar structure (Figure 4b). Conversely, liposomes loaded with VD3, described by other authors, appeared markedly unstable with respect to ET-VD3 and TET-VD3, displaying a 20 nm increase in vesicle mean diameter and decrease in drug content just at day 9 from production [9].

### 2.8. Light Microscopy

Microscopy observations on the uptake and intracellular fate of ET and TET were similar in the three cell types.

**Figure 5:**
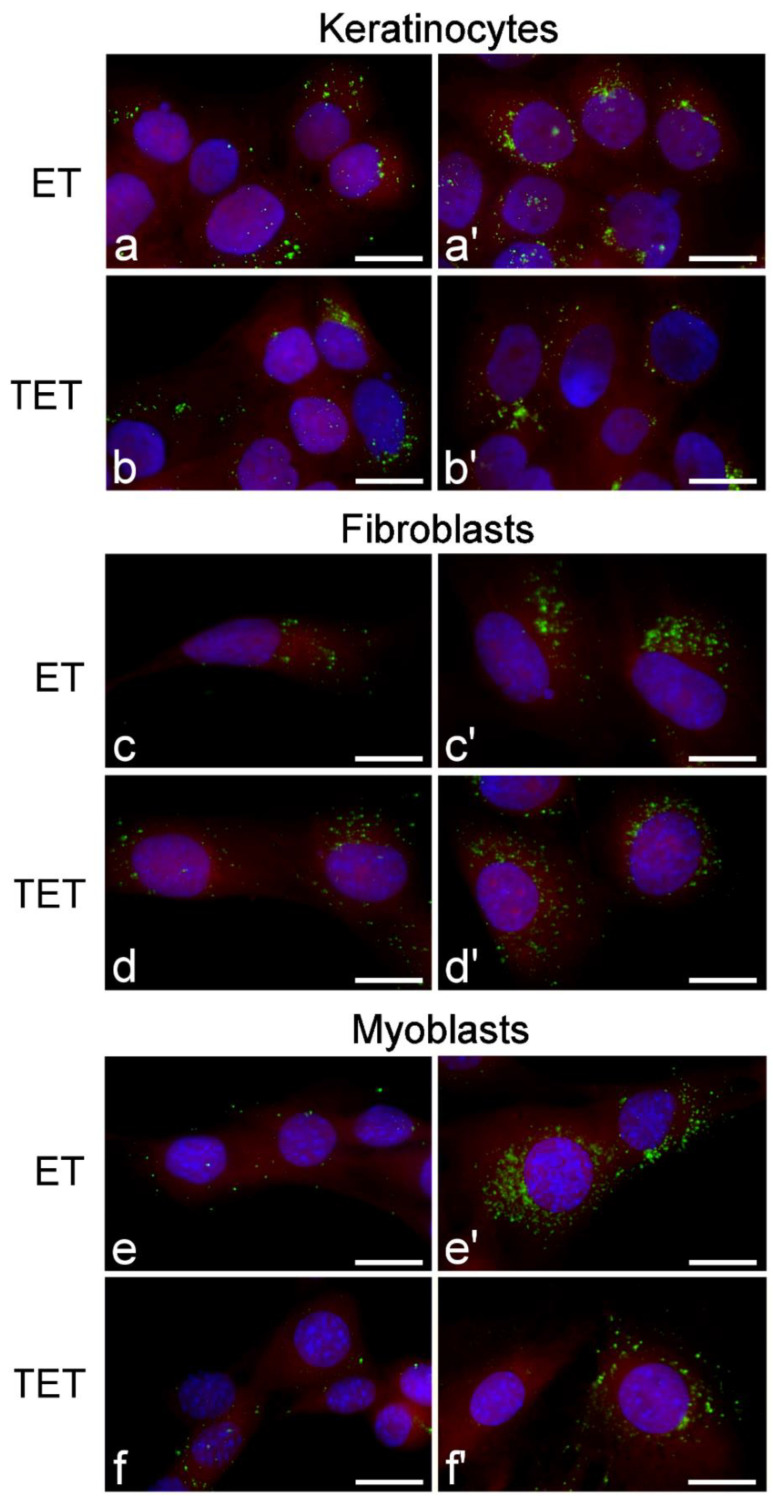
Fluorescence micrographs of keratinocytes, fibroblasts and myoblasts after 2 h (**a**–**f**) and 24 h (**a’**–**f’**) incubation with ET or TET. Nanocarriers in green (PKH67), cytoplasm in red (trypan blue) and nucleus in blue (Hoechst 33342). Bars 20 μm.

At fluorescence microscopy, both ET and TET appeared as green fluorescing spots, while the cytoplasm was counterstained in red and the nucleus in blue (Figure 5). Both ET and TET entered the cells after 2 h incubation, mainly appearing as isolated spots. After 24 h, internalized ET markedly increased in number, while TET did not show evident accumulation. No ET or TET formed large clusters.

**Figure 6:**
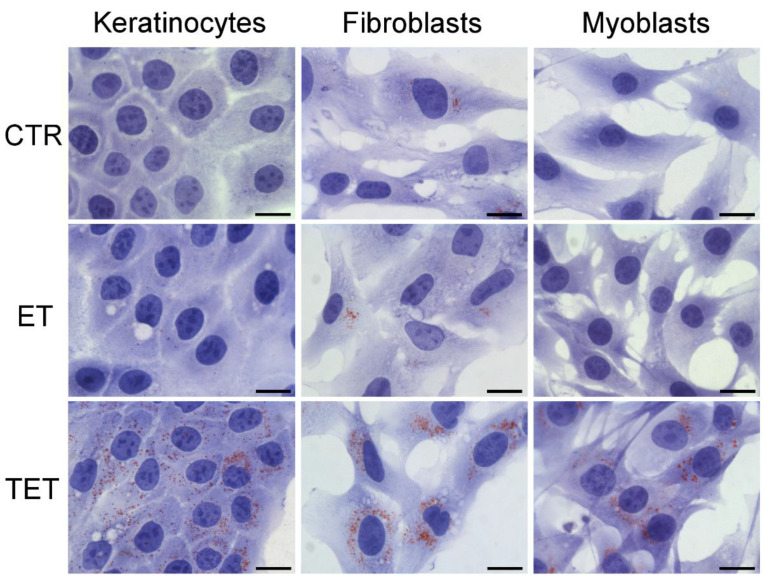
Bright-field micrographs of keratinocytes, fibroblasts and myoblasts after 24 h incubation with ET or TET; CTR are control (untreated) cells. Oil Red O-staining for neutral lipids, hematoxylin counterstaining. Note the marked increase in lipid droplets in TET-treated cells. Bars 20 μm.

Observation of Oil Red O-stained samples at bright-field microscopy (Figure 6) revealed the presence of scarce lipid droplets of small size in all untreated (control) cell types. After 24 h incubation, in cells treated with ET, the amount of lipid droplets remained unchanged, whereas in cells treated with TET, the amount of lipid droplets drastically increased.

### 2.9. Transmission Electron Microscopy

At TEM, ET and TET showed similar behavior in keratinocytes, fibroblasts and myoblasts; therefore, the following description refers to all the cell types.

**Figure 7:**
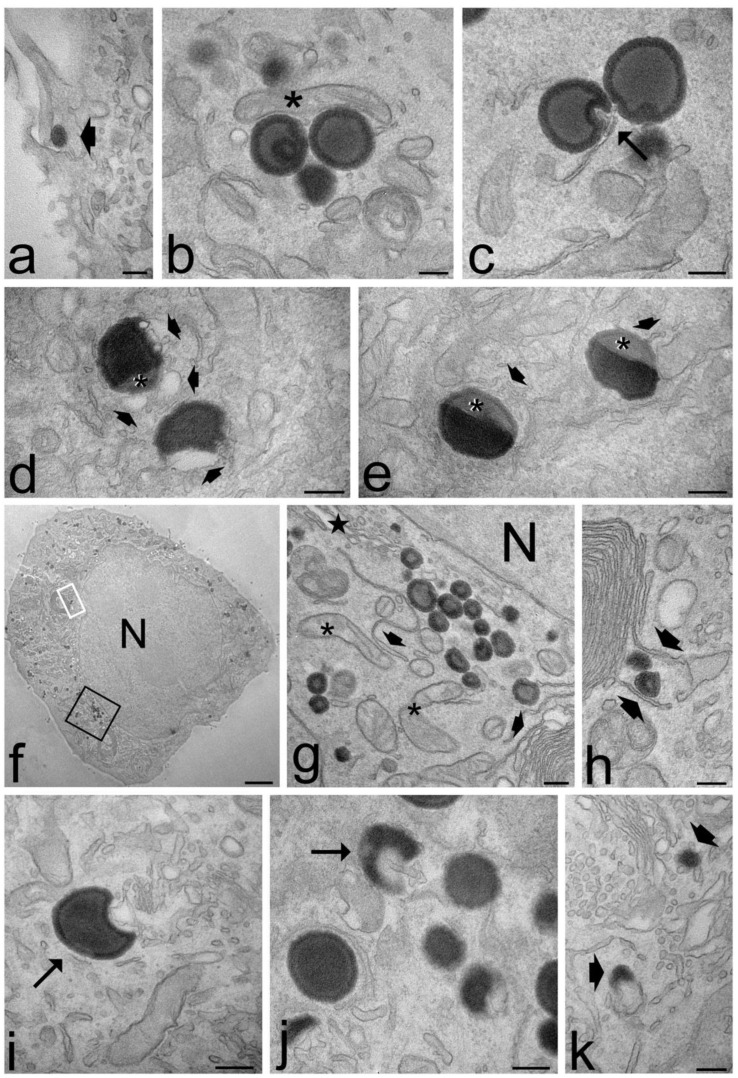
Transmission electron micrographs of keratinocytes (**a**,**d**,**j**,**k**), myoblasts (**b**,**f**,**g**,**h**) and fibroblasts (**c**,**e**,**i**) after 2 h (**a**–**e**) and 24 h incubation (**f**–**k**) with ET. (**a**) An ET (arrowhead) is entering the cell. (**b**) A mitochondrion (asterisk) occurs very close to ET distributed in the cytosol. (**c**) Smooth endoplasmic reticulum into an ET indentation (arrow). (**d**,**e**) ET at various degree of degradation: note the smooth vesicles at their periphery (thin arrows) and the decreased electron density (asterisks). (**f**,**g**) After 24 h incubation, many ET are distributed in the cytoplasm, sometimes very close to the nucleus (N); note the well-preserved morphology of mitochondria (asterisks), endoplasmic reticulum (arrowheads) and Golgi apparatus (star). (**h**) Smooth endoplasmic reticulum cisternae surround two ET (arrowheads). (**g**,**h**) High magnification details corresponding to the black and white framed areas in (**f**), respectively. (**i**,**j**) Crescent-like ET with smooth endoplasmic reticulum inside their concavity. (**k**) ET remnants (arrows) surrounded by many smooth vesicles and tubules. Bars 200 nm (**a**–**e**,**g**–**k**), 2 μm (**f**).

**Figure 8:**
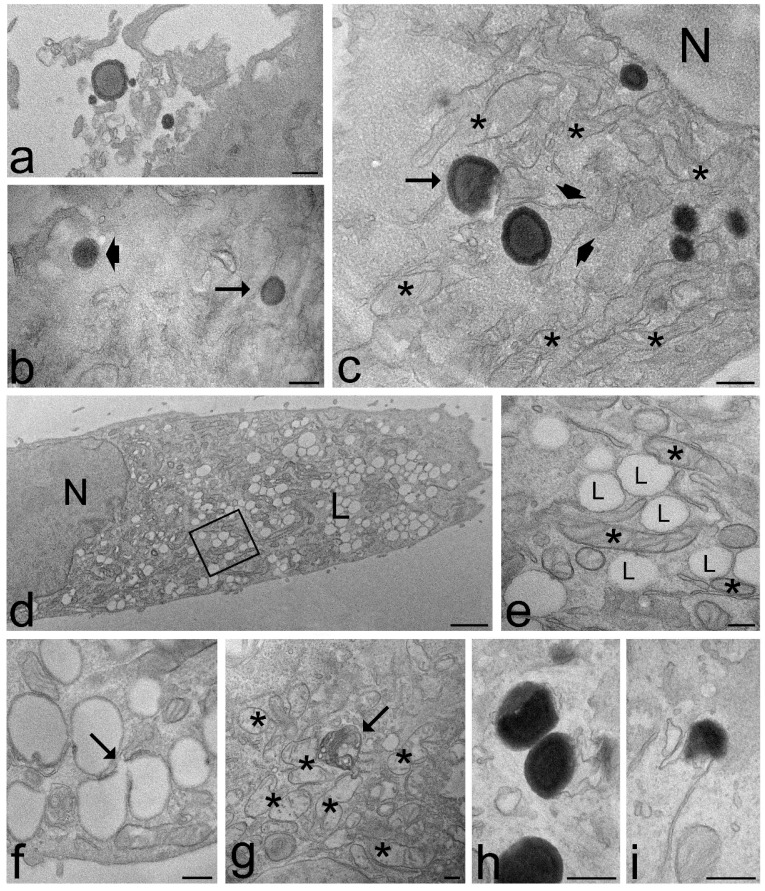
Transmission electron micrographs of keratinocytes (**a**,**f**,**g**,**h**), fibroblasts (**d**,**e**) and myoblasts (**b**,**c**,**i**) and after 2 h (**a**–**c**) and 24 h incubation (**d**–**i**) with TET. (**a**) Some TET occur among microvilli on the cell surface. (**b**) A TET (arrowhead) is entering the cell, while another TET (arrow) occurs free in the cytosol. (**c**) Some TET are distributed in the cytoplasm, sometimes very close to the nucleus (N); note the crescent-like TET (arrow) and the good morphology of mitochondria (asterisks) and endoplasmic reticulum (arrowheads). (**d**,**e**) After 24 h incubation, the cells contain large amounts of lipid droplets (L) of small size; they occur very close each other and are often surrounded by mitochondria (asterisks); (**e**) high magnification detail corresponding to the framed area in (**d**). (**f**) Two coalescing lipid droplets (arrow). (**g**) After 24 h incubation with TET, some mitochondria swell and lose their cristae (asterisks), and residual bodies (arrows) occur. (**h**,**i**) The scarce morphologically recognizable TET show different degradation steps. Bars 200 nm (**a**–**c**,**e**–**i**), 1 μm (**d**).

Both ET and TET appeared as isolated, roundish electron-dense vesicles; when sectioned near their equatorial plane, they showed a dark rim, corresponding to the PC double layer, and a grey core (Figure 7 and Figure 8). No morphological difference was evident between ET and TET after 2 h incubation, but ET showed a larger size than TET (mean diameter ± s.d.: 237.38 ± 6.06 nm vs. 182.17 ± 10.17 nm, respectively; *p* < 0.001, one-way ANOVA test). No statistical comparison was made at 24 h between ET and TET, due to the very limited number of morphologically recognizable TET. ET showed unchanged sizes at 2 h vs. 24 h incubation (237.38 ± 6.06 nm vs. 241.73 ± 11.16 nm, respectively; *p* = 0.842), thus confirming their stability also in the intracellular milieu.

Both ET and TET were found free in the cytosol (Figure 7 and Figure 8a–c); they were ubiquitously distributed in the cytoplasm, from the peripheral to the perinuclear region, but were never found inside the cell nucleus (although some of them occurred very close to the nuclear envelope) (Figure 7g and Figure 8c). Both ET and TET were mostly surrounded by abundant smooth endoplasmic reticulum; some endoplasmic vesicles and cisternae were frequently found to contact the nanocarrier surface and even penetrate into nanocarrier invaginations (Figure 7c–e,i,j and Figure 8c). Some ET and TET took a crescent shape and showed areas of decreased electron density (Figure 7d,e,i,j and Figure 8c,i); in addition, many of them occurred in close proximity of mitochondria (Figure 7b and Figure 8c). ET or TET were never found enclosed in vacuoles, even when occurring very close to the plasma membrane (Figure 7a and Figure 8b).

After 2 h incubation, the intracellular distribution of nanocarriers, their morphology or the cell structural features were similar in cells treated with ET or TET. On the contrary, after 24 h incubation, the intracellular fate of ET and TET appeared strikingly different. ET accumulated in large amounts in the whole cytoplasm maintaining the same morphological features and spatial relationships with cell organelles as observed after 2 h incubation (Figure 7f–h), although some ET remnants were found surrounded by numerous smooth vesicles (Figure 7k). On the other hand, in the cells incubated with TET, only a few morphologically recognizable nanocarriers were observed (Figure 8h,i), while huge amounts of small lipid droplets (mean area ± s.d.: 0.06 ± 0.02 µm^2^) accumulated in the cytoplasm (Figure 8d,e); they were frequently very close to each other, but were only occasionally observed to fuse (Figure 8f). Moreover, many mitochondria were found to border lipid droplets (Figure 8e).

The ultrastructural analysis demonstrated that no morphological alteration or damage of cell organelles occurred after 2 h incubation with for both ET and TET, and after 24 h incubation with ET. Conversely, after 24 h incubation with TET, swollen mitochondria with damaged cristae, and residual bodies were sometimes observed (Figure 8g).

## 3. Discussion

The results of this study demonstrated that ET and TET are potential candidates for the transdermal delivery of VD3, being characterized by suitable size, morphology, deformability and entrapment efficiency [14,15]. These nanocarriers are particularly interesting for two reasons: (i) they are mainly made of PC, the most abundant phospholipid in eukaryotic cells [24], which is a promising pre-requisite for their biocompatibility, (ii) the presence of ethanol confers softness and malleability to ET and TET, and acts as penetration enhancer, promoting their passage through the biological membranes [17,18].

Accordingly, the in vitro cytotoxicity assay revealed that two formulations were safe for all the cell types tested: ET and TET administration did not increase death rate after both short (2 h) and long (24 h) incubation times, up to 86.8 µg PC. Similarly, ET-VD3 and TET-VD3 proved to be mostly safe at the same PC concentrations (the only exception will be discussed below). Checking cell viability and identifying the safe concentrations is essential to evaluate the possible negative impacts of nanocarrier administration since the occurrence of cell death is a main trigger for inflammatory responses [25].

Knowing the internalization mechanisms and intracellular pathways of nanocarriers is also crucial to design efficient delivery strategies [26]. Our combined fluorescence microscopy and TEM analyses provided original information about the uptake and intracellular fate of ET and TET, demonstrating that both efficiently enter keratinocytes, fibroblasts and myoblasts without apparent difference due to cell type. ET and TET occur in the cytoplasm as single units and are unable to enter the cell nucleus: therefore avoiding possible interactions with the nucleic acids and/or nuclear factors that, in a cascade effect, could unpredictably affect nuclear and cellular functions. This observation further confirms the biocompatibility of ET and TET.

Both ET and TET were never observed inside endosomes, even when they occurred close to the plasma membrane; moreover, no plasma membrane invagination typical of early endocytosis was ever found when the nanocarriers were in contact with the cell surface. This suggests that ET/TET cellular uptake does not takes place by classic endocytic processes. ET and TET share chemical and structural similarities with the plasma membrane, thus likely facilitates their interactions: the nanocarriers are made of PC, which is a main phospholipid component of plasma membrane [24], and both nanocarriers and plasmalemma are composed of lipids whose hydrophilic heads are oriented towards water domain, while hydrophobic tails are oriented towards each other, forming bilayers. The easy penetration of ET inside cultured cells has been ascribed to an increase in membrane permeability due to the penetration enhancer effect of ethanol that would, in turn, promote the ET fusion with the plasma membrane and the delivery of ET and loaded molecules inside the cell [27,28]. Our TEM analysis provides unequivocal evidence that both ET and TET maintain their structural integrity after passing through the plasma membrane, as previously observed for liposomes, which, however, rapidly disaggregated once into the cytoplasm [29,30,31]. It may be hypothesized that the presence of ethanol in ET/TET may induce disordering effects in the plasmalemma region making contact with the nanocarrier, loosening lipid packaging [32], thus allowing the passage of these malleable nanocarriers without the typical endosome formation. The uptake mechanism of ET and TET remains unclear, highlighting the need of further research on their interactions with the biological membranes.

Anyway, bypassing the classic endocytic route, ET and TET avoid the rapid degradation due to lysosomal enzymes. However, our ultrastructural observations provided evidence that ET and TET may undergo the action of smooth endoplasmic reticulum-resident enzymes but following distinct intracellular fates. In fact, at short times, both ET and TET occur free in the cytosol, establishing spatial relationships with smooth endoplasmic reticulum and mitochondria. Conversely, at long times, ET mostly preserve their original morphology (which indicates that cell enzymes are unable to massively attack these nanoconstructs), whereas TET are hardly detectable, suggesting that TET are almost completely degraded, while a large amount of lipid droplets accumulate in the cytoplasm, as visualized by both light microscopy cytochemistry and ultrastructural morphology. TEM observations are consistent with the fluorescence images showing an evident accumulation of ET but not of TET after 24 h incubation.

Both ET and TET are made of PC, whose excess in cells is known to be degraded by various enzymes to maintain membrane homeostasis [33,34,35,36]; the main degrading enzyme is phospholipase D that mostly locates in the cell membranes of the smooth endoplasmic reticulum, Golgi complex, endosomes and lysosomes [37]. This explains the close spatial proximity of ET or TET with smooth vesicles and tubules, which were even found penetrating into nanocarrier invaginations. The crescent shape and the concomitant decrease in electron density frequently observed in many ET and TET would indicate a partial nanocarrier degradation. Therefore, the ultrastructural evidence demonstrates that the degradation of both ET and TET occurs through physiological pathways already after 2 h from the uptake (consistent with their safety proved by the cytotoxicity test), but the degradation rate proceeds slowly for ET (which are still present in large amounts after 24 h) while being very fast for TET (which are hardly detectable at TEM after 24 h). The faster degradability of TET is likely due to the presence of T80 that may affect the molecular packing of PC in the bilayer, thus making the TET vesicles more prone to degradation by the phospholipases of the smooth endoplasmic reticulum.

The hydrolysis products of PC include diacylglycerol, phosphocholine, glycerophosphocholine and free fatty acids [33,38]. As high concentrations of free fatty acids are toxic [39], they are incorporated into neutral lipids such as triacylglycerol (a main storage lipid [40]), giving rise to lipid droplets [41,42,43]. Accordingly, numerous lipid droplets were found in cells where massive PC breakdown occurred [43]. Similarly, the massive degradation of TET would release in the cytosol large amounts of PC-derived products, which would accumulate in lipid droplets, whereas the products of the slow degradation of ET would more slowly be released, thus being gradually suitable for multiple metabolic pathways. Moreover, the degradation of T80, occurring only in TET, leads to the formation of free fatty acids and several degradation products including short-chain organic acids [44] that could increase the number of molecules migrating to lipid droplets.

The PC overloading and the consequent lipid droplets accumulation caused by the rapid degradation of TET is likely responsible for some cell stress, as suggested by the presence of altered organelles after 24 h from the treatment. In addition, it is known that T80 degradation gives rise also to peroxides, aldehydes, alkanes, epoxides, ketones [44], which could further increase TET cytotoxicity. Anyway, this stress is evidently unable to affect cell viability even at long term, as demonstrated by the cytotoxicity assay.

Lipid droplets are dynamic organelles able to rapidly change in size and number [45]; they consist of a neutral lipid core of triacylglycerol and cholesteryl ester surrounded by a monolayer mainly composed of PC [46,47] that acts as a surfactant increasing the stability of lipid droplets and preventing their coalescence [48,49]. Accordingly, no large lipid droplets were found in cells treated with TET despite their huge amount: the droplets remained separated even when very close each other and only rare lipid fusions were observed.

PC is also a main component of mitochondrial membranes; however, these organelles lack enzymes for its synthesis, so PC molecules are translocated to mitochondria to be assembled into their membranes [50]. This would explain the frequent finding of mitochondria adhering to the surface of partially degraded ET or TET as well as of lipid droplets.

When ET and TET were loaded with VD3, they did not alter cell viability apart from TET loaded with 25 µm VD3, which increased cell death in myoblasts only after 24 h incubation. All cell types used in this study express enzymes having 25-hydroxylase activity [23,51,52]; accordingly, the administered VD3 did not have toxic effects. However, it has been demonstrated that the administration of high amounts of VD3 can increase cell death in C2C12 cells [52]. TET-VD3 toxicity for myoblasts would be therefore related to the additional stress caused by the massive release of the loaded VD3 in cells already stressed by PC overloading following nanocarrier degradation. Interestingly, after 24 h incubation, VD3 solution induced the highest myoblasts death (about 35%), followed by VD3 administered by TET (about 15%), while VD3 administered by ET was completely safe, demonstrating the capability of nanocarriers to sustain the safe release of high amounts of VD3. This observation further supports the notion that the thorough knowledge of the nanocarrier intracellular degradation pathways is crucial to select the most appropriate formulation strategies for drug-delivery nanoconstructs.

## 4. Materials and Methods

### 4.1. Materials for Ethosome and Transethosome Preparation

Cholecalciferol (VD3), polyoxyethylenesorbitan monooleate, T80, sodium cholate (SC) or dimethyldidodecylammonium bromide (DD) and sodium cholate (SC) were purchased from Sigma-Aldrich (St Louis, MO, USA). Soybean lecithin (PC) (90% PC) was Epikuron 200 from Lucas Meyer (Hamburg, Germany). Solvents were of HPLC grade and all other chemicals were of analytical grade.

### 4.2. Ethosome and Transethosome Preparation 

ET and TET were produced by the “cold method”; briefly, PC was firstly solubilized in ethanol (30% *w*/*v*). Afterwards, bidistilled water was slowly added to the ethanol phase up to a final 70:30 (*v*/*v*) ratio, maintaining magnetic stirring at 750 rpm (IKA RCT basic, IKA^®^-Werke GmbH & Co. KG, Staufen, Germany) for 30 min at 22–25 °C [53]. For TET preparation, the surfactant (i.e., T80 or SC) was solubilized in the PC ethanol solution before water addition. In the case of VD3-containing ET and TET, VD3 (1 mg/mL) was added to PC ethanol solution and rapidly mixed (IKA Vortex 1, IKA^®^-Werke GmbH & Co. KG, Staufen, Germany) before the addition of water. Table 1 reports ET and TET compositions.

### 4.3. Photon Correlation Spectroscopy

Vesicle size analysis of ET and TET was conducted using a Zetasizer Nano-S90 (Malvern Instr., Malvern, England) with a 5 mW helium neon laser and a wavelength output of 633 nm. Measurements were performed at 25 °C at a 90° angle and a run time of at least 180 s. Samples have been diluted with bidistilled water in a 1:20 *v*/*v* ratio. Data were analyzed using the “CONTIN” method [54]. Measurements were performed thrice for 3 months from ET and TET production.

### 4.4. Cryo-Transmission Electron Microscopy

Samples for cryo-TEM were vitrified putting sample droplets (2 µL) for some seconds on a lacey-carbon-filmed copper grid (Science Services, München) [53]. Afterwards, most of the liquid has been removed by blotting paper, obtaining a thin film stretched over the lace holes. The rapid immersion of specimen into liquid ethane cooled to approximately 90 K by liquid nitrogen in a temperature-controlled freezing unit (Leica EMGP, Leica, Germany) instantly allowed their vitrification. All sample preparation steps were conducted at controlled constant temperature in the Leica EMGP chamber. The vitrified specimen was transferred to a Zeiss/Leo EM922 Omega EFTEM (Zeiss Microscopy GmbH, Jena, Germany) transmission electron microscope using a cryoholder (CT3500, Gatan, Munich, Germany). During the microscopy observations, the sample temperature was kept below 100 K. Specimens were examined with reduced doses ≈ 1000–2000 e/nm^2^ at 200 kV. Zero-loss filtered images (∆E = 0 eV) were recorded by a CCD digital camera (Ultrascan 1000, Gatan, Munich, Germany) and analyzed using a GMS 1.9 software (Gatan, Munich, Germany).

### 4.5. Deformability Measurement

The deformability of ET and TET vesicles was determined by extrusion through polycarbonate filter membrane (pore diameter 50 nm). Namely, a stainless steel, 25 mm diameter filter holder (extruder, Lipex Biomembranes, Vancouver, Canada) was employed applying a 2.5 bar pressure at 25 °C and measuring the volume of formulation extruded in 1 min. The mean diameter of vesicles was measured by PCS before and after the extrusion. The deformability of vesicles membrane was calculated according to the following equation:

Def = J x (rv/rp)^2^ (1)

where Def is the vesicle deformability, J is the ratio between the volume of extruded formulation (mL) and the time of extrusion (min); rv is the vesicle size (after extrusion); and rp is the pore size of the filter membrane [15,53].

### 4.6. Vitamin D3 Content of Ethosomes and Transethosomes

The entrapment capacity (EC) of VD3 in ET and TET was determined 1 and 90 days after production. Five hundred microliters of ET and TET were loaded in a centrifugal filter (Microcon centrifugal filter unit YM-10 membrane, NMWCO 10 kDa, Sigma-Aldrich, St. Louis, MO, USA) and ultra-centrifuged (Spectrafuge™ 24D Digital Microcentrifuge, Woodbridge, NJ, USA) at 4000 rpm for 15 min. Afterwards, a 100 μL aliquot of supernatant was diluted with ethanol (1:10, *v*/*v*) and maintained under magnetic stirring for 30 min [18,53]. After filtration of the solution by nylon syringe filters (0.22 μm pores), the amount of VD3 was analyzed by HPLC, as reported below. The EC was determined as follows:

EC = VD3/T_VD3_ × 100 (2)

where VD3 is the amount of drug measured by HPLC and T_VD3_ is the total amount of VD3 employed for ET and TET production.

### 4.7. HPLC Procedure

For HPLC analyses, a two-plungers alternative pump (Agilent Technologies 1200 series, Santa Clara, CA, USA), a UV-detector operating at 325 nm, and a 7125 Rheodyne injection valve with a 50 μL loop were employed. Analyses were conducted eluting a stainless-steel C-18 reverse-phase column (15 × 0.46 cm) packed with 5 μm particles (Platinum C18, Apex Scientific, Alltech, Nicholasville, KY, USA) with a mobile phase containing methanol/water 95:5 *v*/*v*, at a flow rate of 0.6 mL/min. In these conditions, the VD3 retention time was 7.8 min.

### 4.8. Cell Culture and Treatment

HaCaT keratinocytes (an immortalized human cell line purchased from ATCC^®^ PCS-200-011™), fibroblasts (an immortalized human cell line purchased from ATCC^®^ PCS-201-013™) and C2C12 myoblasts (an immortalized murine cell line purchased from ECACC 91031101) were cultured in 75 cm^2^ plastic flasks using Dulbecco’s modified Eagle medium, supplemented with 10% (*v*/*v*) FBS, 1% (*w*/*v*) glutamine, 0.5% (*v*/*v*) amphotericin B, 100 units/mL of penicillin–streptomycin (Gibco, Thermo Fisher Scientific, Waltham, MA, USA) and incubated at 37 °C with 5% CO_2_. Cells were trypsinized in 0.05% EDTA in phosphate buffered saline (PBS) and seeded in flat-bottom 96-well plates (keratinocytes 12 × 10^3^ cells/well; fibroblasts 4 × 10^3^ cells/well; myoblasts 3 × 10^3^ cells/well) for MTT assay or onto glass coverslips (12 mm in diameter) in 24-multiwell (keratinocytes 4 × 10^4^ cells/well; fibroblasts 15 × 10^3^ cells/well; myoblasts 8 × 10^3^ cells/well) for microscopy analyses. Twenty-four hours after seeding, the cells were treated with the nanocarriers for increasing times and then processed according to the specific analysis protocol (see below).

### 4.9. Cytotoxicity Assay

The MTT assay was used to assess the cytotoxicity of ET, TET and DET, as well as of VD3 30% *v*/*v* ethanol solution, ET-VD3 and TET-VD3 on keratinocytes, fibroblasts and myoblasts. The MTT assay is an indicator of cell metabolism and the reduction in absorbance value is related to a loss of oxidoreductase enzyme activity due to the toxicity of the treatment [55]. Cells were treated with blank nanocarrier at different concentrations (from 34.6 to 173.1 μg/mL of PC) selected on the basis of their VD3 entrapment capacity and on VD3 concentrations previously administered in vitro [56]; then, the ET and TET concentrations found to be safe were loaded with VD3 and the MTT assay was applied to ET-VD3 and TET-VD3 in order to verify if drug entrapment changes their safety profile. Cytotoxicity was evaluated after 2 h, in order to detect acute toxic effects, and after 24 h, a longer time than the cell cycle of all cell lines used. Cytotoxicity was not assessed for times longer than 24 h because cell death at such long times may be due to the combined effect of the internalized nanocarriers and the accumulation of toxic products released by the non-internalized nanocarriers undergoing degradation in the medium. Untreated cell samples were used as the control. Some cells were treated with VD3 solution to compare the cytotoxic effect vs. ET-VD3 and TET-VD3 loaded with the same VD3 amounts.

The cytotoxicity assay was performed as follows. At each incubation time, the medium was replaced by 100 µL of MTT solution (Thiazolyl Blue Tetrazolium Bromide, Sigma-Aldrich) (0.5 mg/mL in medium) and incubated for 4 h at 37 °C in a cell incubator. Then, MTT solution was removed, and formazan crystals were dissolved in 100 µL of dimethyl sulfoxide. The absorbance was measured at 570 nm using a ChroMate 4300 ELISA microplate reader (Awareness Technology Inc., Palm City, FL, USA). Experiments were performed in triplicate. Statistical comparisons between the control and experimental conditions were made by the Mann–Whitney pairwise test and significant difference was set at *p* ≤ 0.05.

### 4.10. Light Microscopy

For nanocarriers’ visualization at fluorescence microscopy, ET and TET (i.e., blank nanocarriers) were stained with PKH67 Green Fluorescent Cell Linker Kit for General Cell Membrane Labeling (Sigma-Aldrich) [57]. PKH67 dye was diluted in 500 µL of Diluent C to a concentration of 4 µm (dye solution). Then, 20 µL of ET or TET were diluted in 80 µL of Diluent C and incubated with 100 µL of dye solution (final dye concentration 2 µm) for 5 min while being mixed with gentle pipetting. The reaction was stopped by adding 1 mL of complete medium containing 10% (*v*/*v*) FBS and cells were treated with stained nanocarriers. This technique allowed for the staining of nanocarriers immediately before their use, thus avoiding fluorophore release in the stock ethanol solution. After 2 h and 24 h incubation with either ET or TET, cells were fixed with 4% (*v*/*v*) paraformaldehyde in PBS, pH 7.4 for 30 min at room temperature. Cells were then permeabilized with 0.05% PBS Tween, washed in PBS, incubated with 0.04% Trypan blue (Gibco) in PBS for 30 s, stained for DNA with Hoechst 33342 (0.5 μg/mL in PBS), rinsed in PBS, and finally mounted in 1:1 mixture of glycerol:PBS.

For lipid droplet visualization at bright-field microscopy, the Oil Red O staining for neutral lipids was applied. After 24 h incubation with ET or TET, cells were fixed with 4% (*v*/*v*) paraformaldehyde in PBS, pH 7.4 for 13 min at room temperature. Cells were then rinsed in PBS, incubated with filtered Oil Red O (Bio-Optica, Milan, Italy) for 20 min at room temperature, washed in PBS, stained with Mayer’s Hematoxylin ready-to-use solution (Bio-Optica) for 1 min at room temperature, washed again in PBS, and finally mounted in 1:1 mixture of glycerol:PBS.

The samples were observed with an Olympus BX51 (Olympus Italia Srl, Milan, Italy) microscope using a 40× objective either under bright field mode or in fluorescence (100 W mercury lamp) mode under the following conditions: 450–480 nm excitation filter (excf), 500 nm dichroic mirror (dm), and 515 nm barrier filter (bf), for PKH67; 540 nm excf, 580 nm dm, and 620 nm bf, for trypan blue; 330–385 nm excf, 400 nm dm, and 420 nm bf, for Hoechst 33342. Images were recorded with an QICAM Fast 1394 digital camera (QImaging, Surrey, BC, Canada) and processed using Image-Pro Plus 7.0 software (Media Cybernetics Inc., Rockville, MD, USA). All images were processed using Paint Shop Pro software (JASC Software Inc., Eden Prairie, MN, USA).

### 4.11. Transmission Electron Microscopy

For TEM, control cells and cells treated with ET or TET (i.e., blank nanocarriers) were fixed with 2.5% (*v*/*v*) glutaraldehyde and 2% (*v*/*v*) paraformaldehyde in 0.1 M phosphate buffered saline, pH 7.4, for 2 h at 4 °C, post-fixed with 1.5% potassium ferrocyanide and 1% osmium tetroxide for 1 h, dehydrated with acetone and embedded in Epon resin. In order to preserve the spatial relationships between cells and NPs, both myoblasts and myotubes were processed for TEM as monolayers [58]. Ultrathin sections were observed in a Philips Morgagni transmission electron microscope (FEI Company Italia Srl, Milan, Italy) operating at 80 kV and equipped with a Megaview II camera for digital image acquisition. All images were processed using Paint Shop Pro software (JASC Software Inc., Eden Praire, MN, USA).

Quantitative size evaluation of nanocarriers and lipid droplets was performed by using ImageJ software (NIH). The diameter of 200 nanocarriers per sample was measured in cells treated with ET for 2 h and 24 h, and with TET for 2 h, while the number of TET in cells treated for 24 h was too small for a reliable quantitative evaluation. The sectional area of 200 lipid droplets was measured in cells treated with TET for 24 h.

The mean values ± s.d. of nanocarrier diameter and lipid droplet area were calculated for each time point (2 h and 24 h). Statistical comparisons were performed using one-way ANOVA followed by Dunn’s post hoc test. Significant difference was set at *p* ≤ 0.05.

## 5. Conclusions

Our pilot study demonstrated that both ET and TET are characterized by physicochemical properties optimal for transdermal penetration and efficient VD3 loading, and are safely and easily internalized by cells from epithelial, connective or muscle tissues. Moreover, our detailed ultrastructural study provided original information on the intracellular pathways of ET and TET in cells from these different histological origins. Both nanocarriers are able to intact enter the cells, but they follow distinct intracellular fates: ET persist for long times inside the cytoplasm, without inducing subcellular alteration, while TET undergo rapid degradation, giving rise to an intracellular accumulation of lipids. Therefore, the capability of ET to maintain their structural integrity for long times in the intracellular milieu makes them especially suitable for sustained VD3 release. On the other hand, the rapid intracellular degradation of TET makes them more appropriate for the faster release of VD3.

Based on our results, both ET and TET thus proved to be biocompatible and efficient nanocarriers and may be envisaged as very promising tools for the transdermal delivery of VD3; this paves the way to in vivo study aimed to understand their biodistribution following cutaneous application and to test their therapeutic efficiency in different experimental and pathologic conditions.

## Figures and Tables

**Table 1:** Composition of nanocarriers (*w*/*w*%).

Components	ET	TET	SCET	DET	ET-VD3 ^1^	TET-VD3 ^2^
PC	0.90	0.89	0.89	0.89	0.89	0.90
T80	-	0.3	-	-	0.3	-
SC	-	-	0.1	-	-	-
DD	-	-	-	0.2	-	-
VD3	-	-	-	-	0.1	0.1
Ethanol	29.10	28.81	29.01	28.91	28.80	29.00
Water	70	70	70	70	70	70

^1^ ET loaded with VD3; ^2^ TET loaded with VD3.

**Table 2:** Size distribution parameters of ET and transethosomes, entrapment capacity and deformability of the indicated formulations.

Parameters	ET	TET	SCET	DET	ET-VD3	TET-VD3
Z Average (nm) ^1^	206.3	186.2	276.7	111.2	209.5	246.6
±s.d.	±33	±20	±10	±9	±13	±5
Dispersity index ^1^	0.146	0.131	0.125	0.085	0.136	0.163
±s.d.	±0.00	±0.00	±0.01	±0.02	±0.00	±0.02
EC (%) ^2^	-	-	-	-	100	100
±s.d.					±1.5	±1.0
Def ^3^	6.23	12.55	-	-	16.65	8.74
±s.d.	±0.7	±0.5			±0.3	±0.8

^1^ as determined by PCS; ^2^ entrapment capacity; ^3^ vesicle deformability; s.d.: standard deviation; data are the mean of three independent determinations on different batches.

## Data Availability

Data are contained within the article. Additional data are available from the corresponding author, upon reasonable request.
